# Chicago Medical Society

**Published:** 1872-09-15

**Authors:** Wm. E. Quine

**Affiliations:** Secretary; Chicago


### Society Reports.

### CHICAGO MEDICAL SOCIETY.

Chicago, *Sept.* 2, 1872.

Regular meeting—President Paoli in the chair.

The minutes of the previous meeting were read and approved.

Dr. S. R. Millard reported a case of malignant scarlatina following child-birth, of which the following is an outline:

A lady, aged 27, was delivered of a healthy child, after natural labor, on February 1, 1871. Nothing unusual occurred before the third day after delivery; but at that time the patient vomited once or twice, though her condition was by no means such as would excite apprehension. The following morning she complained of severe headache, but careful examination failed to reveal any other probable cause than an inactive state of the bowels. The tongue was slightly coated, the lochia natural, the pulse 90, respiration natural, and the abdomen neither tumid nor tender. The pulse did not exceed 90 in frequency at any time during the course of the disorder, and was generally below 80. On the morning of the fifth day after delivery the patient said she had rested well during the previous night, and complained of only a very slight headache. The pulse was 80 and rather soft. At 2 o'clock p. m. the pulse was very feeble and intermitting, and there was observed a slight discoloration on the arm and a scarlet rash on the breast. At this juncture ammonia, brandy, and quinine were freely administered, but the patient sank rapidly, and died at 7 p. m.

It was learned, after the diagnosis of scarlatina had been made, that the family which had occupied the house six months before the confinement of the lady, had had the disease, and that the same furniture, bedding, etc., had been used by her, nearly up to the time of confinement, as had been used by the previous occupants of the house. This is the only possible source of contagion of which anything can be learned.

In remarks on the case Dr. Quine stated that he did not think there were present a sufficient number of the ordinary phenomena of scarlet fever to establish beyond doubt the diagnosis. The pulse and temperature described were by no means such as are ordinarily met with in the disease, and the entire absence of local complications would throw additional doubt on its existence. The patient was not known to be in an alarming condition until the fifth day after delivery, when an eruption appeared, and simultaneously the circulation became greatly depressed. He inquired if this was usually the case in scarlatina, and stated as his opinion that the condition of the circulation generally improved in that disease immediately upon the appearance of the eruption. It was, moreover, reasonable to suppose that poison which had been in the system during a period of over six months without apparent detriment would have caused to be developed a certain degree of tolerance; at least would not manifest such violent action as to destroy life in four or five hours after the appearance of the first symptom that would indicate its presence. From the history that had been read he considered it as reasonable to suppose that the patient died of puerperal fever, and the eruption as petechial, as to regard the case as an easily recognizable one of scarlatina.

An animated discussion then ensued on the nature and forms of puerperal fever, between Drs. Wickersham, Paoli, and Quine.

The President, Dr. Paoli, reported a case of poisoned wound, in which severe constitutional symptoms followed the bite of a species of tarantula, said to be abundant in the vicinity of the city. Soon after the wound was inflicted there appeared around it a dark erysipeloid rash ; and on the following day a similar rash appeared on the opposite and uninjured limb.

Dr. Bridge regarded the last mentioned occurrence as an example of reflex action. He did not intend to say that a specific morbid or remedial impression could be conveyed through the medium of the nervous system, but that a poison might so impress a sensitive part as to cause such a condition of the nervous center as would result in an influence being transmitted to another part as would lower its vitality or power of resistance.

Dr. Quine reported a case of multilocular ovarian tumor, and exhibited the specimen, which was removed post mortem. The patient, when she came under the doctor's care, was fifty-six years of age, and in an excellent condition of general health. She had ceased menstruating ten years before, and noticed at that time a small swelling or tumor in the right inguinal region. For over nine years the tumor did not cause any inconvenience, and did not seem to increase in size; but shortly after her arrival in this country from Norway, nine months ago, it began to enlarge rapidly, and when she presented herself for treatment the abdomen was so enormously distended as to embarrass very seriously the action of the heart and lungs. She was tapped at once, and over a gallon of limpid serum drawn off. Refusing to submit to the radical operation at this time, she was dismissed from care after her recovery from the tapping, with a prescription for iron and quinine. In a month, however, she presented herself again, and readily consented to an operation; but a few days before the time set for performing it she was injured by a fall from a chair, in consequence of which death from peritonitis ensued. The tumor weighed about twenty-two pounds. The pedicle was rather short and broad. There had been no adhesion.

WM. E. QUINE, *Secretary.*

SMALL-POX IN THE UTERUS.—Traces of variolous eruption have been discovered in fœtal life at four months, which is the earliest period on record.—*Med. Rec.*

